# Letrozole Monotherapy in Pre- and Early-Pubertal Boys Does Not Increase Adult Height

**DOI:** 10.3389/fendo.2019.00201

**Published:** 2019-04-05

**Authors:** Tero Varimo, Sanna Toiviainen-Salo, Taneli Raivio, Liisa Kerttula, Leo Dunkel, Matti Hero

**Affiliations:** ^1^Pediatric Research Center, Children's Hospital, Helsinki University Hospital, Helsinki, Finland; ^2^Department of Pediatric Radiology, HUS Medical Imaging Centre, Helsinki University Hospital, University of Helsinki, Helsinki, Finland; ^3^Faculty of Medicine/Physiology, Medicum, University of Helsinki, Helsinki, Finland; ^4^Department of Musculoskeletal Radiology, HUS Medical Imaging Centre, Helsinki University Hospital, University of Helsinki, Helsinki, Finland; ^5^Barts and the London School of Medicine and Dentistry, William Harvey Research Institute, Queen Mary University of London, London, United Kingdom

**Keywords:** idiopathic short stature, letrozole, adult height, long-term follow-up, MRI

## Abstract

**Background:** Aromatase inhibitors (AIs) have been used in boys with idiopathic short stature (ISS) to promote growth despite the lack of actual data regarding treatment effect on adult height. In this study, we characterized adult heights and long-term follow-up in AI-treated boys with ISS.

**Methods:** Adult heights and long-term follow-up data, including spine MRIs, of a randomized, double-blind, placebo-controlled trial of boys who were treated with letrozole (Lz) (2.5 mg/d) or placebo (Pl) for 2 years during prepuberty and early puberty. The mean bone ages at treatment cessation were 10.2 and 10.8 years, respectively.

**Results:** Adult heights were similar between the boys treated with Lz (*n* = 10) and those who received Pl (*n* = 10) (164.8 ± 4.0 vs. 163.7 ± 3.7 cm, *p* = 0.49, respectively). In either group, the adult heights did not differ from predicted adult heights at start of the study [Pl: 163.7 (3.7) cm vs. 166.9 (3.3), *p* = 0.06; Lz: 164.8 (4.0) cm vs. 167.6 (7.9), *p* = 0.20, respectively]. Long-term follow-up data showed that the frequency of subjects with a vertebral deformity was similar between the groups (Lz, 29% and Pl, 22%, *p* = 0.20), and no single comorbidity was clearly enriched in either group.

**Conclusions:** The Lz-treated boys had similar adult heights with the subjects who received Pl for 2 years, which indicates that the treatment is not beneficial when given to pre- or early-pubertal boys. Previously observed vertebral deformities ameliorated during follow-up, which supports the skeletal safety of Lz therapy in children and adolescents.

## Introduction

Third generation aromatase inhibitors (AIs), such as letrozole (Lz) and anastrozole, are compounds that effectively suppress estrogen biosynthesis and have been shown to delay bone maturation in boys with growth hormone deficiency (GHD), constitutional delay of growth and puberty (CDGP), and idiopathic short stature (ISS) (1–4). The treatment with AIs has resulted in an increased predicted adult height (PAH) after 1–3 years of treatment (1, 3, 5, 6). These promising results have prompted the interest of using AIs for growth indications in adolescent boys (7, 8), albeit the long-term efficacy of the treatment on height gain lacks verification, since adult height data from controlled trials of AI-treated boys is limited to one study that included 6 boys with CDGP who received Lz for 1 year during mid-puberty (9). Furthermore, the long-term safety of blocking estrogen biosynthesis during childhood is unclear (7), particularly regarding the influence of AI-treatment on skeletal health (9). The concerns that long-term use of AIs might be detrimental to spine vertebral morphology stem from our previous report that shows an increased risk of mild vertebral deformities of unknown clinical significance in boys treated with Lz prior to the onset of puberty (9).

The short-term efficacy and safety data of AI-treatment in adolescents are reassuring (1, 3, 4), but the availability of long-term data is extremely limited. In fact, the lack of evidence has resulted in confusion and differing views on the off-label use of such treatment in growth indications, varying from relatively liberal use in pubertal short boys to strictly limited pediatric use in clinical studies pending long-term efficacy and safety results from randomized controlled trials (7, 8, 10, 11).

In this study, we report adult heights and long-term follow-up data, including spine health assessed with magnetic resonance imaging (MRI), in boys with ISS who were treated with either Lz or placebo (Pl) for 2 years during prepuberty or early puberty.

## Materials and Methods

The study population included 30 pre- and early-pubertal boys with ISS who participated in a randomized, double-blind, placebo-controlled study, in which the boys were treated with either Lz (2.5 mg/d, *n* = 16) or Pl (*n* = 14) for 2 years in an attempt to increase adult height (1). When the study started, little was known about the optimal timing of the treatment in this target population, and prepubertal boys who were expected to progress in puberty in the near future were permitted to participate. For this study, the boys were contacted once more and asked to participate in a final study visit in order to measure adult heights and to evaluate long-term effects of the treatment.

Seventeen of the 30 boys (57%), 8 of whom were treated with Lz and 9 with Pl, agreed to come to the adult height visit, and were evaluated in the pediatric endocrinology outpatient clinic at the Helsinki University Hospital (HUH) between 2012 and 2017. Additionally, we included the height measurements of three boys (two with Lz and one with Pl) who were at near adult height at the post-treatment follow-up visit (9). These boys had bone ages of more than 17 years, and, consequently, had used over 98% of their growth potential (12). Thus, the overall number of patients with adult height or near adult height was 20 (67%, *n* = 10 in each group). All the boys in the Pl group and eight boys in the Lz group were prepubertal at the beginning, and, after the treatment, twelve of the 20 boys (seven with Pl and five with Lz) were pubertal. Baseline characteristics and distribution of Tanner genital stages at the beginning of the study and at the treatment cessation are shown in Table 1. The mean bone ages at treatment cessation were 10.2 and 10.8 years in Lz and Pl groups, respectively. Predicted adult heights were calculated by the Bayley-Pinneau method (12), which produces a percentage of height attained relative to final height.

**Table 1:** Characteristics of the participants with adult height or near adult height data.

	**Lz (*n* = 10)**	**Pl (*n* = 10)**
**AT THE START OF THE STUDY**		
Height (cm)	129.7 (7.9)	127.5 (7.5)
Height (SDS)	−2.4 (0.3)	−2.5 (0.4)
Age (years)	11.5 (1.8)	10.9 (1.8)
Bone age (years)	9.2 (2.6)	8.7 (1.9)
Testicular volume	1.5 (1.4)	1.0 (0.6)
Testosterone level (nmol/l)	1.4 (1.9)	0.4 (0.4)
**AT THE ADULT HEIGHT VISIT**		
Height (cm)	164.8 (4.0)	163.7 (3.7)
Height (SDS)	−2.6 (0.7)	−2.7 (0.7)
Age (years)	23.3 (4.0)	21.7 (3.1)
Bone age (years)	18.5 (0.7)	18.7 (0.7)
Testicular volume (ml)	12.8 (3.0)	12.2 (3.5)
BMI (kg/m^2^)	22.1 (3.2)	23.8 (5.4)
PAH at the start of the study (cm)	167.6 (7.9)	166.9 (3.9)
PAH after 2 years of treatment (cm)	174.0 (8.3)	167.4 (4.3)^a^^*^
Change in PAH during treatment (cm)	6.4 (2.2)	0.5 (4.4)^b^
Bone age after 2 years of treatment (years)	10.2 (2.9)	10.8 (1.5)
Tanner G-stage of puberty at the start of the study, distribution from G1 through 5, (n)	8/2/0/0/0	10/0/0/0/0
Tanner G-stage of puberty at treatment cessation, distribution from G1 through 5, (n)	5/0/1/1/3	3/3/2/2/0

*Mean (SD)*.

a *p = 0.049*,

b *p = 0.004. P-values are for between group comparisons*.

* *The difference remained significant in the subgroup of 17 boys with complete skeletal maturation [Lz: 174.9 (9.1), Pl 167.5 (4.5), p = 0.047]. The difference remained significant in the subgroup of 17 boys with complete skeletal maturation [Lz: 6.8 (2.3), Pl 0.1 (4.5), p = 0.002]. PAH, predicted adult height by Bayley-Pinneau (12)*.

At the adult height visit, weight was measured with a stabilized and calibrated scale (Seca® 878dr) and height with a calibrated, wall-mounted Harpender stadiometer with 0.1 cm precision or, in minority of patients (*n* = 5), portable stadiometer (Seca® 213). Subjects were interviewed, and chronic diseases and medication were recorded. Pubertal stage was determined and testicular volume (ml) was calculated by using the formula: length (cm) × width^2^ (cm) × 0.52 (13). In all boys, puberty was complete as evaluated with Tanner genital and pubic hair stages (14). In addition, we reviewed the medical records of the 30 boys who participated in the original study and collected information regarding co-morbidities that had required medical attention (1). Mid-parental target height was calculated by using the formula: 0.886 × [(father's height + mother's height)/2 + 6.8–178.9066]/6.6784–0.071.

Spine health was re-evaluated by MRI in 12 subjects at the adult height visit. Additionally, we included the MRI findings of four patients from the post-treatment visit, since these boys had had bone age of more than 17 years (9). Spine MRIs were performed with a 1.5 T imager (Intera Achieva, Philips Medical Systems, Best, Netherlands) using a total-spine imaging protocol and analyzed as described before (9). In detail, special interest was paid on vertebral body anatomy, endplates, disk height, and muscle status. The MRI images were evaluated by experienced radiologists (ST-S and LK), who were blinded to the treatment modality. At the adult height visit, vertebral anatomy was classified by using the classification for pediatric vertebral body morphology (15), since it enabled the comparison of the results to the MRI findings from the post-treatment visit (9). In detail, vertebral anterior and middle wedge deformities were classified as mild (grade 2a) if the reduction in anterior height was between 20 and 50%, and severe (grade 2b) if it was more than 50%. Compression deformities were classified into mild (grade 3a) and severe (grade 3b), if the reductions in anterior, middle, and posterior height were between 20 and 50% or >50%, respectively. The back muscle status was evaluated by measuring the average psoas muscle thickness (mm).

The study was reviewed and approved by HUH and the Ethics Committee of HUH, and a written informed consent was obtained from all the participants. The study was performed according to the Declaration of Helsinki.

### Statistical Analyses

The data were analyzed by using SPSS statistical software for Windows, version 22.0 (SPSS, Chicago, IL, USA). The results are presented with mean (standard deviation) unless otherwise mentioned. The comparisons between the two groups were performed with Mann-Whitney U-test and with Fisher's exact test. Adult or near adult heights were compared with PAHs at the beginning of the study (1) and at the post-treatment visit (9, 15) and with target heights by using Wilcoxon signed-rank test. Spearman rank correlations were calculated. Number needed to harm (NNH) with Lz-treatment was calculated for the risk of vertebral deformity. Statistically significant level was set to *p* < 0.05.

## Results

### Adult Height

**Figure 1:**
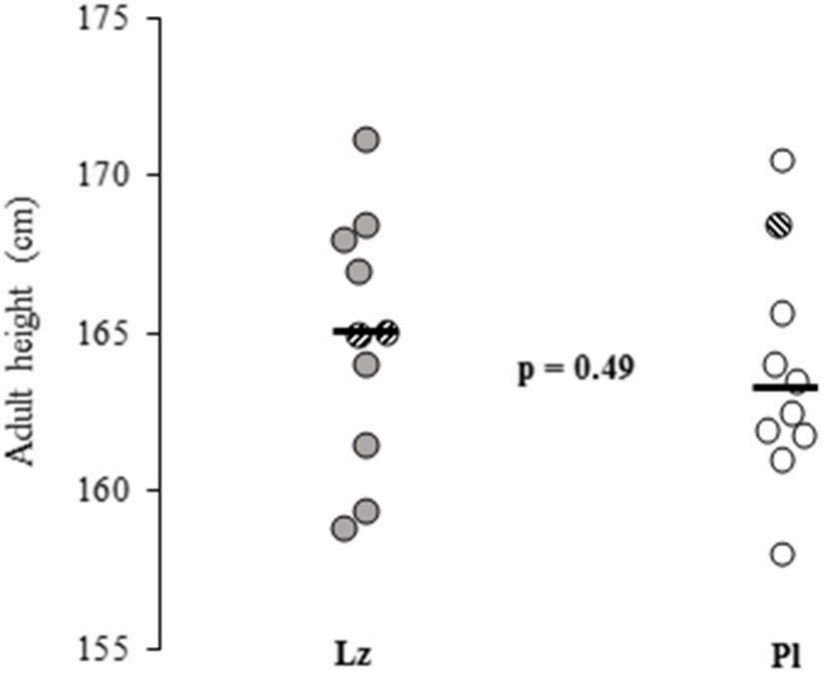
Adult heights and near adult heights (striped circles) of 20 boys with idiopathic short stature who were treated with letrozole (Lz) or placebo (Pl) for 2 years during pre and early puberty. Horizontal black lines represent means and the *p*-value shows the between group comparison significance. Lz, letrozole; Pl, placebo.

The clinical and auxological data did not differ between the treatment groups and are detailed in Table 1. The boys who were treated with Lz (*n* = 8) had similar adult heights with the subjects who received Pl (*n* = 9) [164.7 (4.6) vs. 163.2 (3.5) cm, *p* = 0.47, respectively]. In addition, when comparing the boys who had reached adult height or near adult height (*n* = 20), no differences were found between the groups [Lz: 164.8 (4.0) and Pl: 163.7 (3.7) cm, *p* = 0.49] (Figure 1). The changes between adult height SDS or near adult height SDS and height SDS at the beginning of the study were also similar between the groups [Lz: −0.22 (0.8) and Pl: −0.27 (0.7), *p* = 0.97].

Then, we compared the adult height or near adult height to the PAH at the beginning of the study. In both groups, the adult heights were similar to the PAHs at start of the study [Pl: 163.7 (3.7) cm vs. 166.9 (3.3), *p* = 0.06; Lz: 164.8 (4.0) cm vs. 167.6 (7.9), *p* = 0.20, respectively]. The difference between adult height or near adult height and PAH at the beginning was similar between the Lz [−2.8 (5.1) cm] and the Pl groups [−3.2 (4.5) cm, *p* = 0.65]. Further, in both groups, adult or near adult height SDSs were significantly lower than the target heights [Lz, *n* = 10: −2.0 (0.6) SDS, *p* = 0.005 and Pl, *n* = 8: −2.4 (0.5) SDS, *p* = 0.012], and no difference in adult or near adult height minus target height was found between the groups [−2.0 (0.6) vs. −2.4 (0.5) SDS, *p* = 0.32]. In the Lz group, only five boys progressed in puberty during the treatment, and had similar adult height or near adult height with those who remained prepubertal (*n* = 5) [164.3 (4.0) vs. 165.5 (4.5) cm, *p* = 0.63, respectively]. Moreover, the difference between adult height or near adult height and PAH at the beginning was similar between pubertal Lz-treated boys [0.5 (1.9) cm] and the Pl group [−3.4 (4.9) cm, *p* = 0.098]. In the Lz group, the boys who progressed in puberty had more advanced bone age than the Pl-boys at the end of the treatment [Lz; 12.9 (0.4) vs. 11.5 (1.3) years, *p* = 0.028]. Of note, bone ages at the start of the study did not correlate with adult or near adult heights (r_s_ = −0.12, *p* = 0.62).

### Vertebral Morphology

Five mildly deformed vertebral bodies were found in three patients, two in Lz and one in Pl group (Table 2). The frequency of vertebral deformities, intervertebral disk and endplate abnormalities were similar between the groups (Table 2). Then we compared the MRI findings to the results from the post-treatment visit (9). Of the five boys with Lz who had vertebral deformities detected before (9), three were re-evaluated with MRI at the adult height visit. In one Lz-treated boy, the previously reported vertebral deformity was visible in the adult height visit MRI, whereas the previously reported mild deformities (2a and 3a) in the other two Lz-treated boys had normalized. Interestingly, one patient with Pl had developed a mild (grade 3a) deformity in Th5 vertebral body. The absolute risk increase of Lz-treatment for vertebral deformity was 17.5% (95% CI −21.8 to 56.7) and NNH was 6. Both treatment groups had similar average psoas muscle thicknesses [Lz: 42.1 (5.9) mm and Pl: 41.4 (5.9) mm, *p* = 0.81]. Two Pl-treated boys reported mild exercise-related lumbar back pain.

**Table 2:** Vertebral morphology in 16 boys with idiopathic short stature at completion or near completion of growth (bone age over 17 years) as evaluated by MRI.

	**Lz (*n* = 7)**	**Pl (*n* = 9)**	*P*-value^*^
Patients with vertebral deformity (n)	2	1	0.55
Deformed vertebrae (n)	4	1	0.32
Grade 2a	2	1	
Grade 2b			
Grade 3a	2		
Grade 3b			
Patients with endplate abnormalities (n)	2	4	0.63
Endplate abnormalities (n)	15	30	
Patients with intervertebral disk abnormalities (n)	2	2	0.92

*Deformed vertebrae are classified based on the classification for pediatric vertebral body morphology (15)*.

* *P-values are for between group comparisons analyzed with Fisher's exact test*.

### Comorbidities

Seven of the 30 boys had a comorbidity that had required medical attention. Comorbidities included depression, psychosis, chronic fatigue syndrome, ulcerative colitis, and operated maxillary hypoplasia. Interestingly, 5 of the 7 patients had a psychiatric disease and antidepressant (*n* = 3) or antipsychotic (*n* = 2) medication. The frequency of subjects with psychiatric comorbidity, however, did not differ significantly between those who were treated with Lz (*n* = 4) and those who received Pl (*n* = 1, *p* = 0.34).

## Discussion

In this follow-up study of our previously conducted placebo-controlled randomized trial (1), we found that the pre- and early-pubertal boys, who were previously treated for 2 years with an AI, had similar adult heights with those who received Pl. The boys treated with Lz had approximately 1.1 cm higher adult heights than the subjects treated with Pl, which is neither statistically nor clinically relevant. Thus, since this is the first report on the impact of AI therapy on adult height in short boys in a placebo-controlled randomized setting, the hard evidence that would support the long-term growth-promoting effects of aromatase inhibition is still lacking. This is a disappointment, since previous RCTs have shown that treatment with 3rd generation AIs effectively delays bone maturation and increases PAH by 5.1–6.7 cm during 1–3 years of treatment in boys with ISS, CDGP, or GHD, while allowing puberty to continue at a normal rate (1, 2, 4). Based on these findings, the off-label use of AIs in growth indications has expanded despite the lack of evidence on long-term efficacy (i.e., adult height gain). Our results indicate that, in boys with ISS, the beneficial effect of a potent AI monotherapy given during pre- or early-puberty is attenuated by later growth and maturation. This, however, does not rule out a beneficial effect of AI-therapy on adult height if the intervention is targeted exclusively to pubertal boys, and the treatment is continued until late stages of puberty. Only studies that follow subject to adult height will reveal the effectiveness of AI- and other treatments aimed at increasing adult height. To this end, in boys with CDGP, a short 12-month co-treatment with Lz resulted in higher adult height with borderline statistical significance than the treatment with testosterone alone (4, 9). Further, when compared with GH alone, 36 months of combined therapy with anastrozole and GH significantly increased PAH in boys with GHD, who were followed up until late stages of puberty with a mean bone age of 17.2 years (2). Both of these studies, however, suffered from significant dropout rates during follow-up, which increases the risk of attrition bias. A recent study in boys with ISS, which compared AI, GH, and their combination, reported favorable near adult heights (mean bone age of 15.3 years) in subjects treated with the combination (3). However, this study had no placebo-treated control group, and no statistical comparison of near adult height between AI plus GH and GH monotherapy groups, which hampers drawing further conclusions on the impact AI therapy on height outcome.

Our results on spine MRIs are reassuring and suggest that 2 years of AI-treatment in prepuberty or early puberty is not detrimental to spine health in the long-term. We reported previously that mild vertebral body abnormalities were detected in five out of 11 (45%) participants assigned to Lz, whereas there were none in the Pl group (9). This raised the concern that Lz might disturb the normal growth and development of vertebral bodies. Further, the deformities were found only in boys who received Lz before the onset of puberty. In this study, we showed that these deformities had normalized in two out of three subjects with available long-term follow-up data. At completion of growth (i.e., bone age above 17 years), significant differences in vertebral parameters between the groups were no longer evident. At the same time, we acknowledge that our study has a small sample size, and a larger samples size might yield a statistical significance. Our finding is in line with previous studies with anastrozole and supports the view that aromatase inhibitor treatment does not increase the risk of vertebral deformities in the long-term (3). Additionally, we found that endplate deformities were equally frequent in both groups (16). This is in agreement with the current knowledge that endplate deformities are a relatively common finding among adolescents as ~5% of healthy adolescents have these changes in spine MRI (17).

We found no significant difference in the frequency of comorbidities between the Lz and Pl groups. Altogether, 22% of the boys had an ascertained comorbidity that required medical attention. Interestingly, the Lz-treated boys had higher frequency (25%) of psychiatric comorbidity than the subjects with Pl (7%), but the difference did not reach statistical significance. To our knowledge, AI treatment in males has not been associated with psychiatric morbidity. In primates, Lz easily penetrates the blood-brain barrier (18), and there is evidence that in males AI treatment may modulate cognitive capacity and increase risk-taking behavior (19). In Lz-treated boys of this study, the treatment had no measurable impact on cognitive performance (20), which supports the view that AI treatment appears not be harmful to adolescent cognitive development.

Aromatase inhibition removes the negative feedback of estrogen on the hypothalamic-pituitary-gonadal axis, which results in stimulated FSH secretion and advanced testicular growth (1, 21). Testicular size is known to correlate well with sperm count (22). On the other hand, aromatase knockout mice develop impairment of spermatogenesis, and in men with congenital aromatase deficiency semen samples have ranged from normal to oligospermia (23, 24). Our finding that the Lz-treated subjects had similar adult testicular volumes with their peers with Pl supports the view that AI-treatment during pre- and early-puberty had no detrimental influence on spermatogenesis. In line with this, a previous study found that boys with GHD previously treated with AI had similar sperm concentration, motility, and morphology to their healthy peers (25). Future larger studies evaluating semen samples of AI-treated boys are warranted.

Several patients were lost during the follow-up resulting in limited sample size which may result in attrition bias and influence the interpretation of the results. However, this is the largest RCT to describe adult height in AI-treated boys and our dropout rate is comparable to that of other RCTs (2, 9). Arguing against significant attrition bias, the participants with available adult height data had a similar increase in PAH during treatment with the total population of Lz-treated boys (1). It is of note that the PAH produced by Bailey-Pinneau method over-estimated adult height by about 3 cm. This is a documented feature of the method in boys with retarded bone age [reviewed in (26)].

## Conclusions

This study reported similar adult heights between patients who were previously treated with Lz and those who received Pl, which questions the adult height-promoting effect of pre- and early-pubertal AI-monotherapy in boys with ISS. As half of the patients on Lz remained prepubertal during the treatment, our findings may not be generalizable to short boys treated until late stages of puberty. A larger placebo-controlled RCT, targeted to pubertal short boys, is needed to establish the efficacy of AI therapy in promoting adult height. Lz-treatment had no long-term detrimental association on spine vertebral morphology, which supports the skeletal safety of AI therapy in boys with ISS.

## Author Contributions

TV collected and analyzed data, drafted the initial manuscript, and reviewed and revised the manuscript. ST-S designed the study, analyzed the radiological data, and reviewed and revised the manuscript. LK analyzed the radiological data, reviewed, and revised the manuscript. TR analyzed data, reviewed, and revised the manuscript. MH designed the study, designed the data collection instruments, coordinated and supervised data collection, carried out the initial analyses, reviewed, and revised the manuscript. LD conceptualized and designed the study, reviewed, and revised the manuscript. All authors approved the final manuscript and agree to be accountable for all aspects of the work.

### Conflict of Interest Statement

The authors declare that the research was conducted in the absence of any commercial or financial relationships that could be construed as a potential conflict of interest.
